# Anticancer polymers designed for killing dormant prostate cancer cells

**DOI:** 10.1038/s41598-018-36608-5

**Published:** 2019-01-31

**Authors:** Haruko Takahashi, Kenji Yumoto, Kazuma Yasuhara, Enrico T. Nadres, Yutaka Kikuchi, Laura Buttitta, Russell S. Taichman, Kenichi Kuroda

**Affiliations:** 1 Department of Biologic and Materials Sciences, School of Dentistry, University of Michigan, Ann Arbor, MI 48109 USA; 2 Department of Biological Science, Graduate School of Science, Hiroshima University, 1-3-1 Kagamiyama, Higashi-hiroshima, Hiroshima, 739–8526 Japan; 3 Department of Periodontics & Oral Medicine, School of Dentistry, University of Michigan, Ann Arbor, MI 48109 USA; 4 Division of Materials Science, Graduate School of Science and Technology, Nara Institute of Science and Technology, Ikoma, Nara, 630–0192 Japan; 5 Molecular, Cellular and Developmental Biology, University of Michigan, Ann Arbor, MI 48109 USA

**Keywords:** Pharmaceutics, Biopolymers, Bioinspired materials

## Abstract

The discovery of anticancer therapeutics effective in eliminating dormant cells is a significant challenge in cancer biology. Here, we describe new synthetic polymer-based anticancer agents that mimic the mode of action of anticancer peptides. These anticancer polymers developed here are designed to capture the cationic, amphiphilic traits of anticancer peptides. The anticancer polymers are designed to target anionic lipids exposed on the cancer cell surfaces and act by disrupting the cancer cell membranes. Because the polymer mechanism is not dependent on cell proliferation, we hypothesized that the polymers were active against dormant cancer cells. The polymers exhibited cytotoxicity to proliferating prostate cancer. Importantly, the polymer killed dormant prostate cancer cells that were resistant to docetaxel. This study demonstrates a new approach to discover novel anticancer therapeutics.

## Introduction

The design and development of molecules effective in eliminating dormant cancer cells remain a significant challenge in drug discovery. Dormant cancer cells are not proliferating but are largely resistant to conventional chemotherapies which target only rapidly proliferating cells, leading to recurrence and metastasis^1,2^. The design of drugs which are able to target dormant cancer cells must meet at least two conditions: (i) the target mechanism/pathway and molecular target in cancer cells are not dependent on cell proliferation, and (ii) the molecular target must be distinctive from normal cells for selectivity. To that end, we hypothesize that cell membranes would serve as an anticancer target in dormant cancer cells to meet these requirements. Specifically, physically compromising the barrier function of cell membrane would be lethal, but not necessarily be dependent on the stages of cell cycle. For the second requirement, cancer cells overexpress phosphatidylserine (PS) on their cell membrane surfaces relative to normal cells, which could provide a cancer selective binding target^3,4^. Therefore, designer molecules capable of binding to PS lipids and disrupting cell membranes may effectively kill dormant cancer cells.

In this study, we report the design of new class of anticancer polymers that are effective in killing dormant prostate cancer (PCa) cells. The design is inspired by membrane-active host-defense peptides with anticancer activity^5–7^. This class of peptides, which we denote as anticancer peptides (ACPs), are relatively small (2,000–5,000 Da) and adopt cationic amphiphilic α-helical structures. Cationic ACPs selectively bind to PS lipid-rich anionic cancer cell membranes preferentially to normal cells by electrostatic interactions. The bound ACPs insert the hydrophobic domain of their helix into the membranes, which causes membrane disruption, leakage of cellular components, and ultimately cancer cell death. The anticancer polymers used here are low molecular weight methacrylate copolymers with random-sequences of binary monomer compositions consisting of cationic and hydrophobic side chains, serving as a model platform to test our approach. We hypothesize that the ACP-mimetic polymers are selective to PCa cells and kill dormant PCa cells resistant to conventional anticancer drugs. Synthetic polymer platforms are more resistant to proteolytic degradation in physiological environments and are more chemically tunable for activity modulation than peptides. The anticancer activity of natural and synthetic cationic polymers has been previously examined and were shown to induce apoptosis in cancer cells^8,9^. However, the molecular mechanism is not clear, and thus it is difficult to control the activity by design or develop structure-activity relationships. In addition, many synthetic polymers used in anticancer therapeutics are simply drug delivery carriers in the forms of drug conjugates or drug-encapsulated particles where the anticancer activity originates primarily from the drugs, not the polymers^10–12^. Recently, Yang and Hedrick demonstrated cationic polymer assemblies with anticancer activity by lysing cancer cell membranes^13^. The membrane-targeting mechanism would be a new strategy to treat drug-resistant cancer cells.

## Results and Discussion

### Polymer design and synthesis

**Figure 1:**
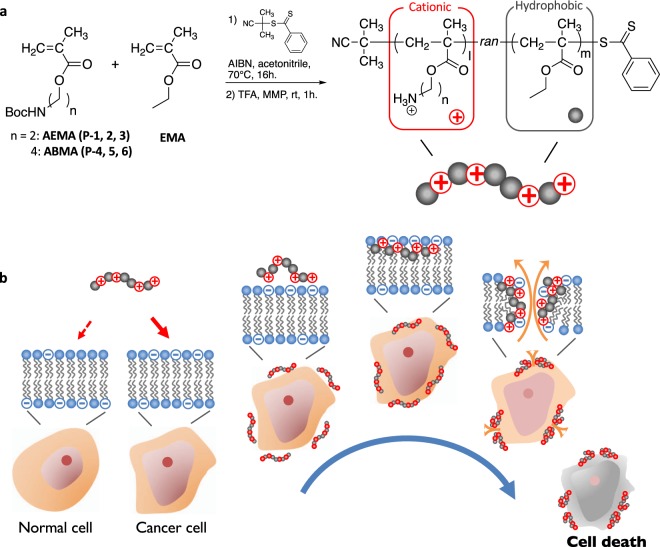
Synthesis and proposed mechanisms of anticancer copolymers. (**a**) Synthesis of methacrylate random copolymers with cationic and hydrophobic side chains. The polymers with low molecular weights (~2,000 g/mol.) and narrow molecular weight distribution were prepared by RAFT controlled radical polymerization. The composition of hydrophobic monomer EMA was varied. (**b**) The proposed anticancer mechanism of polymers.

Learning from ACPs, our first hypothesis is that the cationic and hydrophobic properties of ACPs are the essential functionalities, but also the minimum requirements that govern their membrane-targeting anticancer activity and cancer cell-selectivity. To test the design hypothesis, we prepared methacrylate random copolymers consisting of cationic and hydrophobic side chains (Figs 1a and S1). To identify the optimal compositions, a series of cationic amphiphilic random copolymers were synthesized (Tables 1 and S1). The primary ammonium group was chosen to mimic the cationic lysine side chains of ACPs, which should bind to the anionic PS lipids by electrostatic interaction, imparting selectivity to cancer cells over human cells (Fig. 1b). The ethyl side chains of ethyl methacrylate (EMA) monomers serve as the hydrophobic groups for insertion into the cell membranes, causing membrane disruption. The molecular weights of polymers are 2,000 to 3,000 g/mol., in a similar range to the molecular size of ACPs^5^, and the molecular weight distributions of the polymers are relatively narrow (dispersity *Đ* = 1.1–1.3) (Table 1). The polymers are highly soluble in both water and cell culture media.

**Table 1:** Characterization of anticancer peptide-mimetic copolymers.

Polymer	Composition	DP^a^	*M*_*n*_^b^ (NMR) (g/mol.)	*M*_*n*_^c^ (GPC) (g/mol.)	*M*_*w*_^c^ (GPC) (g/mol.)	*M*_*w*_/*M*_*n*_^c^
**P-1**	AEMA100 - EMA0	15	2,200	2400	2700	1.15
**P-2**	AEMA72 - EMA28	13	1,800	1800	2000	1.13
**P-3**	AEMA60 - EMA40	13	1,800	1700	1900	1.10
**P-4**	ABMA100 - EMA0	14	2,400	2200	2900	1.32
**P-5**	ABMA69 - EMA31	16	2,500	2800	3100	1.12
**P-6**	ABMA52 - EMA48	17	2,500	2100	2400	1.12

^a^The degree of polymerization was calculated by comparing the integrated areas of signals from the side chains to that from the phenyl group (RAFT agent) at the polymer end.

^b^The number average molecular weight (*M*_*n*_) was calculated from the degree of polymerization (DP), and molecular weights of monomers and chain transfer agent. The molecular weight of trifluoroacetate was excluded in *M*_*n*_ in order to compare the size of copolymers to that of ACPs.

^c^*M*_*n*_ and *M*_*w*_ were determined using GPC for boc-protected polymers.

### Cytotoxicity against proliferating prostate cancer cells vs. normal cells

**Figure 2:**
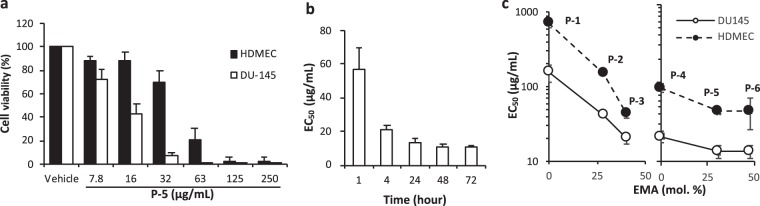
Cytotoxicity of copolymers to prostate cancer cells and HDMEC. **(a**) Cytotoxicity of **P-5** to DU145 and human dermal microvascular endothelial cells (HDMEC) after 24 hour incubation at 37 °C. The percentage of cell viability after 24 hour treatment was calculated relative to the viability of cells treated by a vehicle (0.01% acetic acid, the final concertation of 0.001% in the assay cell culture.) for the copolymer as 100%. (**b**) Time dependence of EC_50_ of **P-5** for DU145. The EC_50_ values were determined from dose-response curves of each incubation time. (**c**) Relationship between the hydrophobic EMA monomer composition and anticancer activity. The EC_50_ values of all polymers for DU145 cells after 24 hour treatment with the copolymers were plotted against the mol. percentage of EMA in a polymer chain. The data points and error bars for panels represent average value ± standard deviation (S.D.) of three independent assays, performed in triplicate for each (n = 3).

To test our design approach, the copolymers were first examined for their *in vitro* cytotoxicity to proliferating cancer cells. We selected three established metastatic PCa cell lines C4-2B, DU145, and PC-3 which are associated with bone tropism and brain metastasis (C4-2B and DU145) and bone metastasis (PC-3)^14^. The copolymers caused concentration-dependent cytotoxicity in these PCa cells and exhibited 100% killing in the culture at high polymer concentrations (Figs 2a and S2). The EC_50_ value, the concentration of copolymers that gives half-maximal reduction in cell viability, decreased significantly for the first 4-hour incubation with **P-5** (Figs 2b and S3 for dose-cell viability curves), but leveled off for longer incubation times. This indicates that the copolymers act relatively quickly, in contrast to docetaxel that generally needs several days to be effective *in vitro*. The EC_50_ values after 24 hour treatment varied from 10 to 500 µg/mL, depending on the cell lines and monomer compositions (Table 2). In general, the polymers were more active (lower EC_50_ values) against C4-2B and DU145 than PC-3 cells. The EC_50_ values decreased as the mole percentage of EMA monomer increased (Fig. 2c), indicating that the hydrophobicity of polymers potentiates the anticancer activity due to the role of hydrophobic groups causing membrane disruption. The copolymers with longer cationic side chains (ABMA monomers) showed higher activity (smaller EC_50_ values) compared to those with shorter cationic side chains (AEMA monomers). We have previously demonstrated that the elongation of cationic side chains of methacrylate random copolymers increases the depth of polymer insertion in anionic bacterial cell membranes (snorkeling effect), leading to higher antibacterial activity^15^. Similarly, the longer cationic side chains might enhance the polymer insertion into cancer cell membranes and disrupt the membranes more effectively. As a reference, the EC_50_ values of lytic peptide melittin were 2–6 µg/mL under the same assay conditions. While melittin exhibited higher activity than the polymers, it is highly toxic to normal cells, thus limiting its use as an anticancer agent^16,17^.

**Table 2:** Cytotoxicity of copolymers to prostate cancer cells and non-cancerous cells.

Entry	EC_50_^24 h^ (µg/mL)						HC_50_ (µg/mL)
	Human prostate cancer cells			Human primary cells			Human red blood cells
	C4-2B	DU145	PC-3	HDMEC	NHDF	HPrEC	
**P-1**	207 ± 33	157 ± 42	457 ± 21	700 ± 82	n.d.	n.d.	(2.8 ± 1.0%)^†^
**P-2**	46.7 ± 9.6	42.0 ± 1.6	113 ± 32	153 ± 5	45.3 ± 4.1	28.0 ± 5.0	(11.7 ± 9.3%)^†^
**P-3**	39.6 ± 6.2	20.0 ± 3.3	70.7 ± 7.4	43.3 ± 4.7	n.d.	n.d.	137 ± 95
**P-4**	18.7 ± 3.8	21.0 ± 3.7	54.7 ± 10.5	86.0 ± 9.9	13.3 ± 5.3	16.5 ± 0.5	(0.9 ± 0.2%)^†^
**P-5**	18.7 ± 1.9	13.3 ± 2.5	30.7 ± 3.4	43.0 ± 2.4	9.3 ± 2.5	11.0 ± 1.0	(26.9 ± 9.6%)^†^
**P-6**	20.0 ± 1.6	13.7 ± 2.6	27.3 ± 1.9	44.3 ± 19.4	n.d.	n.d.	71.3 ± 23.0
Melittin	6.3 ± 0.2	1.8 ± 0.2	2.8 ± 0.4	3.5 ± 0.3	n.d.	n.d.	2.7 ± 0.8

The cell viability was measured by a colorimetric cell viability assay CCK-8 or XTT and the EC_50_ values were determined from the dose-cell viability curves (Fig. S2) after incubation with the copolymers for 24 hours. The data points and error bars represent average value ± standard deviation (S.D.) of three independent assays, performed in triplicate (n = 3).

^†^The percent hemolysis at 1,000 µg/mL is presented in parentheses.

The cytotoxicity of copolymers to non-cancerous normal cells was also evaluated to determine selectivity. Lysis of human red blood cells (hemolysis) was used as a measure of membrane-mediated acute cytotoxicity of copolymers. In general, the hemolytic activity of copolymers depended on their hydrophobicity. The percent hemolysis increased as the mole percentage of EMA increased, indicating that the copolymers with higher hydrophobicity are more hemolytic (Table 2 and Fig. S4 for dose-hemolysis curves). The cytotoxicity of copolymers to proliferating normal cells after 24 hour exposure to the copolymers was first examined using human dermal microvascular endothelial cells (HDMECs) (Fig. 2a and Table 2). The viability of HDMECs after incubation with **P-5** for 24 hours was higher than that of similarly treated DU145 cells. Similar to DU145, the EC_50_ values for HDMECs also decreased as the mole percentage of EMA increased (Fig. 2c). These results suggest that the high hydrophobicity of polymers led to undesired cytotoxicity against normal human cells, that is likely due to non-specific hydrophobic binding to normal cell membranes.

We further examined the cytotoxicity of copolymers against primary human cells including normal human dermal fibroblasts (NHDF) and human prostate epithelial cells (HPrECs). The EC_50_ values after 24 hour incubation with the selected copolymers were smaller than those against HDMEC and PCa cells (Table 2 and Fig. S2), indicating that the copolymers showed higher cytotoxicity against these primary human cells as compared to HDEMC and PCa cells. These results suggest that the cytotoxicity of copolymers to primary normal cells is largely dependent on the cell types.

### Membrane-disruptive mechanism

**Figure 3:**
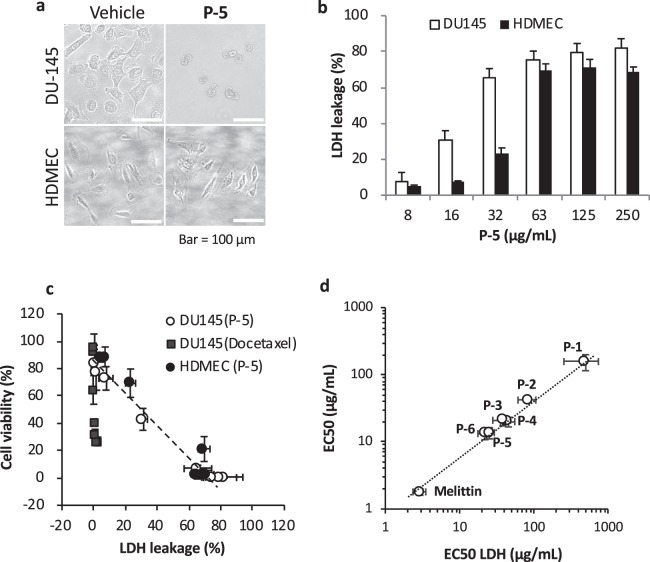
Membrane-targeting action of copolymers. (**a**) Effect of **P-5** on cell surfaces of DU145 and HDMEC cells. The cells in a 6-well plate were incubated with **P-5** for 24 hours at concentration of 32 µg/mL, and the cell morphologies were observed. 0.01% acetic acid was used as vehicle (the final concertation of 0.001%). (**b**) Polymer-induced LDH leakage from DU145 cells and HDMEC. The cells in a 96-well plate were incubated with **P-5** for 24 hours. Each assay was performed in triplicate, and the data points and error bars represent average value ± standard deviation (S.D.) of three independent assays (n = 3). (**c**) Correlation between the percentages of cell viability and LDH leakage for DU145 cells treated with **P-5**. (**d**) Correlation between the EC_50_ values for the cell viability reduction and for the LDH leakage by **P-5** after incubation with **P-5** for 24 hours.

To probe the mechanism of action by the copolymers, we further examined the effect of **P-5** on the cell membranes. We selected **P-5** as a model copolymer because it showed a potent activity against PCa cells and relative selectivity to PCa cells over HDMECs among the polymers tested in this study. We first observed the DU145 cells and HDMECs under a microscope after incubation with **P-5** for 24 hours (Fig. 3a). Because of cell death, DU145 cells were detached, and the remaining cells were collapsed. The surfaces of these cells were severely damaged. On the other hand, the cell surfaces of HDMECs treated with **P-5** for 24 hours appear to be little damaged compared to those of DU145. To quantify the damages to the cell membranes, an extracellular leakage of the cytoplasmic enzyme lactate dehydrogenase (LDH) from DU145 cells treated with **P-5** for 24 hours was examined. **P-5** caused significant LDH leakage at high polymer concentrations, suggesting that the cell membranes were permeabilized (Figs 3b and S5). The percentage of released LDH was correlated to cell viability of cells treated by **P-5** (Fig. 3c) and other copolymers (Fig. S6). On the other hand, the conventional anticancer drug docetaxel, which interferes with cell division of rapidly proliferating cells and causes apoptotic cell death^18^, caused little or no LDH release although it reduced cell viability (Figs 3c and S5–S7). In addition, the EC_50_ values of all the polymers for the cell viability and LDH leakage were well correlated (Fig. 3d). These results suggest that the polymer-induced cancer cell death was primarily caused by cell membrane disruption.

We also determined damages to the cell membranes of HDMEC by **P-5** after 24 hour treatment. **P-5** also caused LDH leakage (Fig. 3b), which indicate that **P-5** also cause membrane disruption in the HDMEC cell membranes. The level of LDH leakages was smaller than that of DU145, which appears to reflect the selectivity of **P-5** to DU145 over HDMECs in their cell viabilities (Fig. 2a). Similar to DU145 cells, the percentage of released LDH was also correlated to the cell viability of HDMECs treated by **P-5** for 24 hour exposure to **P-5** (Fig. 3c). This result suggests that the cytotoxicity of **P-5** to HDMECs was also primarily caused by cell membrane disruption. However, the LDH leakage was suppressed (leakage ~20%) after 72 hour incubation at high **P-5** concentrations (Fig. S8) while these cells showed no viability (Fig. S9). We speculate that the cells might form junctions and seal the damaged membranes, but we cannot rule out other mechanisms to cause toxicity in cells, which involve cellular targets. At this point, the toxicity mechanism of copolymers is not clear, which is beyond the scope of this report.

### The role of PS lipids in the cytotoxicity mechanism of copolymers

**Figure 4:**
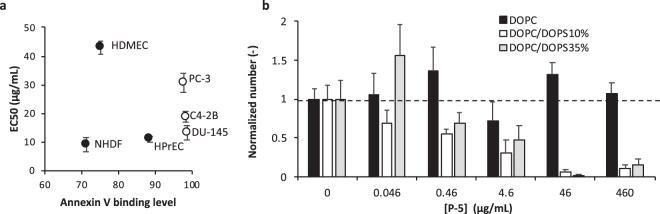
Role of PS lipids in the anticancer mechanism of P-5. (**a**) The relationship between the EC_50_ values and Annexin binding level. (**b**) Lysis of GUVs consisting of DOPC/DOPS (65:35 or 90:10) or only DOPC by **P-5**. The number of GUVs with diameters larger than 5 nm, relative to those of control (0 µg/mL), was determined as a measure of **P-5** induced lysis. The data points and error bars represent average value ± standard deviation (S.D.) of 4 or 5 images.

The results described above indicated that the cytotoxicity of copolymers depends on cells, and the copolymers appear not to be selective to cancer cells over normal cells. We wondered if these normal cells might also express significant PS lipids on the cell surfaces, and then the cytotoxicity of copolymers might be correlated to the level of PS lipid exposure on the surfaces of these cells. To test this hypothesis, we examined the PS lipid exposure of these cells using Annexin V. Annexin V is a biomarker which binds specifically to PS lipids on cell surfaces, and the Annexin V binding to cells was further analyzed by flow cytometry (Fig. S10). To quantify the level of Annexin V binding to cells, we gated regions which give higher than the high end of 5% in the cell population without Annexin V (control) for each cell type, and the percentage of cells stained by Annexin V in these gates was determined. The PCa cells all displayed high Annexin V binding levels (~98%), as compared to the primary normal cells NHDF (71%) and HDMEC (75%), while HPrEC (89%) showed a relatively high level of Annexin V binding. The EC_50_ values of cell viability was plotted as a function of level of Annexin V binding (Fig. 4a), and there appears to be no direct correlations, indicating that the cytotoxicity of copolymers may not directly reflect the level of PS lipid exposure on the cell surfaces.

Based upon our results we next wondered what roles do PS lipids have in regulating the anticancer activity of copolymers? The results of LDH leakage (Fig. 3) suggested that the copolymers acted by disrupting cancer cell membranes. Here we examined the ability of **P-5** to target and disrupt a lipid bilayer with PS lipids. We prepared giant unilamellar vesicles (GUVs) with (DOPC/DOPS) or without PS lipids (DOPC). Lipid vesicles have been previously used to probe the mechanism of anticancer peptides^19–21^. It has been reported that the total amount of PS lipids in cells were largely different in cancer cell types, ranging from 2% to 10%^22^. In order to examine the effect of PS lipids on the copolymer activity evidently, we used GUVs with 10% PS lipids, which would be potentially the maximum amount of PS lipids exposed on the cancer cell surfaces. **P-5** completely lysed all GUVs with PS lipids at high **P-5** concentrations, although the GUVs without PS lipids remained intact (Figs 4b and S11), indicating that the PS lipids enhanced the membrane disruption by **P-5**. The selective activity of **P-5** against the membranes with PS lipids can be explained by the electrostatic binding of cationic side chains of **P-5** to anionic PS lipids. Therefore, the PS lipids could be the first binding target. However, increasing the amount of PS lipids from 10% to 35% did not enhance the lysis of GUVs (Fig. 4b). In addition, it has been also reported that phosphatidylethanolamine (PE) lipids are also externalized to the cancer cell surfaces^23^. The anticancer peptide Polybia-MP1 lysed GUVs containing PE more than those without PE, while PE lipids are zwitterionic, but not anionic, indicating that the PE lipids play an important role in the membrane-disruptive mechanism of the peptide. This suggests that lipids other than PS lipids and/or their combinations with PS lipids may be important for membrane disruption by the copolymers. Taken together, the PS lipids may be involved as an initial binding site in the membrane disruptive mechanism of copolymers, but they are not only the factor to determine the activity of copolymers against cell membranes. This may corroborate with our conclusion on no clear correlation between the cytotoxicity (EC_50_) of copolymers and the level of Annexin binding (Fig. 4a). In addition, detailed mechanistic studies would be also need to directly prove that the copolymers indeed target PS lipids on the actual cell surfaces. One potential approach would be use of lacadherin which binds to PS lipids and blocks the effect of polymers by preventing the interactions with PS lipids^24^, which will be a subject of our future study.

### Anticancer activity against dormant cancer cells

**Figure 5:**
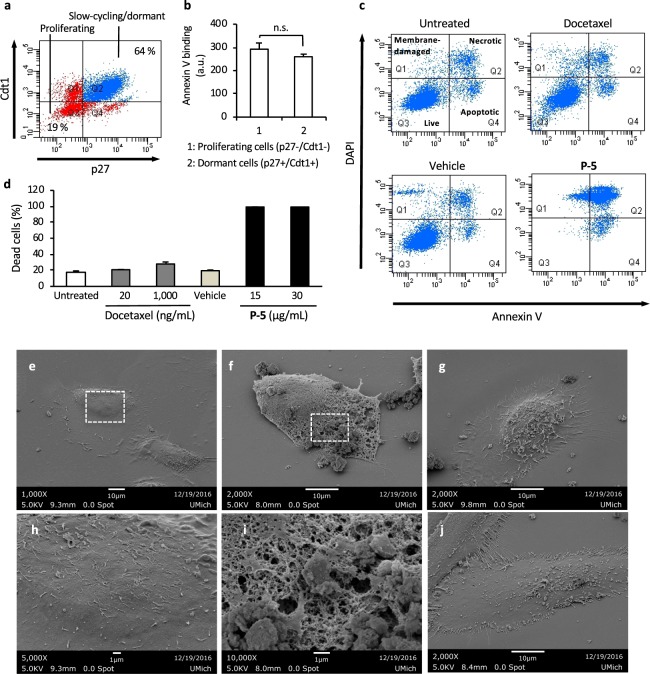
Anticancer activity of **P-5** against isolated dormant PC-3 cells. (**a**) Cell populations. The cells were cultured in a 0.5% FBS medium for 48 hours, and the sub-populations of cells in different cell cycles were identified by fluorescence signals associated with the reporters p27 and Cdt1 in flow cytometry. (**b**) Levels of PS exposure on proliferating and dormant PC-3 cells. (**c**) Flow cytometric analysis of treated isolated dormant PC-3 cells. The dormant PC-3 cells were incubated with **P-5** at 30 µg/mL, docetaxel at 20 ng/mL, or vehicle (0.001% acetic acid) for 48 hours in a 0.5% FBS culture medium. (**d**) Cytotoxicity of **P-5** to dormant PC-3 cells. The percent cell death is calculated as 100%- live cell subpopulation (DAPI(−)/Annexin V(−)). Each group was measured with three replicates (n = 3), and the error bars indicate the standard deviation. (**e**–**j**) Effect of copolymer **P-5** on dormant PC-3 cells observed by SEM. The isolated dormant PC-3 cells were untreated (**e,h**) or treated with **P-5** at 30 µg/mL (**f,i**), docetaxel at 20 ng/ml (**g**), or vehicle (0.01% acetic acid, the final concertation of 0.001%) (**j**) for 1 hour in a 0.5% FBS culture medium. The panels of (**d**,**e**) are magnified images of areas indicated by broken lines in the panels of (**h**,**i)**, respectively.

We next examined the efficacy of **P-5** to kill dormant cancer cells. We used an *in vitro* cellular model prepared under low serum conditions to induce dormant cell behavior. In general, when cells were cultured in a reduced serum medium, the cell cycle becomes slow or arrested, inducing cell dormancy^25^. However, the cell viability of PC-3 cells still increased after being cultured in a 0.5% FBS medium for 48 hours, indicating that some sub-populations of cells were proliferating (Fig. S12). To examine the cell cycle and to isolate dormant cells, we integrated cell cycle reporters (p27 and Cdt1) into PC-3 cells, which report the stage of cell cycle by fluorescence^26^. The reporters-integrated PC-3 cells were cultured in a 0.5% FBS medium for 48 hours. The dormant cells (p27(+)/Cdt1(+)) made up 64% of the total cell population, while the actively proliferating cells (p27(-)/Cdt1(-)) were 19% (Fig. 5a). The dormant cells were sorted by flow cytometry and cultured in a 0.5% FBS medium. The dormancy of the isolated cells was confirmed by low expression of the endogenous proliferation marker Ki-67 protein^27^ (Fig. S13) and no substantial increase in the cell proliferation over 48 hours in the same culture medium (Fig. S14). Importantly, the dormant and proliferating cells showed the same level of Annexin V-APC binding (Fig. 5b), suggesting that anionic PS lipids exposed on the cell surfaces are viable targets for both proliferating and dormant PC-3 cells.

The isolated dormant PC-3 cells were treated with docetaxel or **P-5** for 48 hours. The cell viability-based assay was not suitable to determine their cytotoxicity due to the low viability readings from dormant cells. Therefore, their effect on treated cells was determined by flow cytometric analysis using DAPI and Annexin V-APC, which report membrane-damaged cells and apoptotic cells, respectively^28^. DAPI diffuses into the cytosol and nucleus of cells with damaged cell membranes and bind to DNA. Annexin V binds to the elevated level of PS lipids on apoptotic cell surfaces. It should be noted that the fluorescence signals from Annexin V bound to apoptotic cells were typically one or two orders of magnitude higher than those from Annexin V bound to viable cancer cells. In the case that cells are doubly positive, the cell membrane integrity is significantly compromised (necrotic), in which PS lipid exposure is no longer regulated and/or Annexin V freely binds to PS lipids in the inner leaflet of the cell membranes.

The flow cytometry analysis indicates that docetaxel caused little or no changes in the dormant PC-3 cells as compared to the untreated control (Fig. 5c). The percent cell death of docetaxel-treated dormant cells was 20%, which was not significantly different from 17% dead cells of control (Fig. 5d). The extremely high concentration of docetaxel (1,000 ng/ml) increased dead cells only slightly to 28%. These results suggest that docetaxel was inherently inactive against the dormant PC-3 cells. On the other hand, the cells treated with **P-5** were stained by both DAPI and Annexin V, suggesting significant membrane damage or necrotic cell death. At the **P-5** concentrations of 15 µg/mL and 30 µg/ml, in which the cell viabilities of HDMECs were larger than 70% (Fig. 2a), ~100% of the dormant PC-3 cells were dead (Fig. 5d). These results support our hypothesis that the ACP-mimetic copolymer is capable of killing dormant cancer cells.

To further probe the anticancer mechanism of the copolymer, we treated the cells with **P-5** for 1 hour because we anticipated that early stage apoptosis or intermediate membrane damage can be detected in flow cytometry analysis. The flow cytometry results showed that only double positive and double negative cell sub-populations were observed (Fig. S15), indicating that **P-5** caused deleterious damages in the cell membranes without any intermediate steps of membrane permeabilization or apoptosis. The cell surfaces of dormant PC-3 cells treated with **P-5** for 1 hour were visualized by electron microscopy and showed large pores or a sponge-like morphology (Fig. 5e–j), along with many pieces of small debris around the cells. However, the cells treated with docetaxel and vehicle remained intact. The cells also appear to partially adhere to the substrate. These results indicate that **P-5** caused significant membrane damage in a relatively short timeframe.

### Copolymer activity against dormant cells in a solid tumor model

Within solid tumors, some cell sub-populations located far from blood vessels are metabolically inactive or dormant due to depletion of oxygen and nutrients^29,30^. These dormant cells do not respond to anticancer drugs which target proliferating cancer cells. It is also known that poor penetration of drug molecules into solid tumor tissues also reduces their efficacy^31,32^. As the copolymer effectively killed dormant cancer cells in monolayer cultures, we further tested the copolymer for the activity against those in an *in vitro* tumor spheroid model, which provides more clinically relevant tumor-like microenvironment^33^.

**Figure 6:**
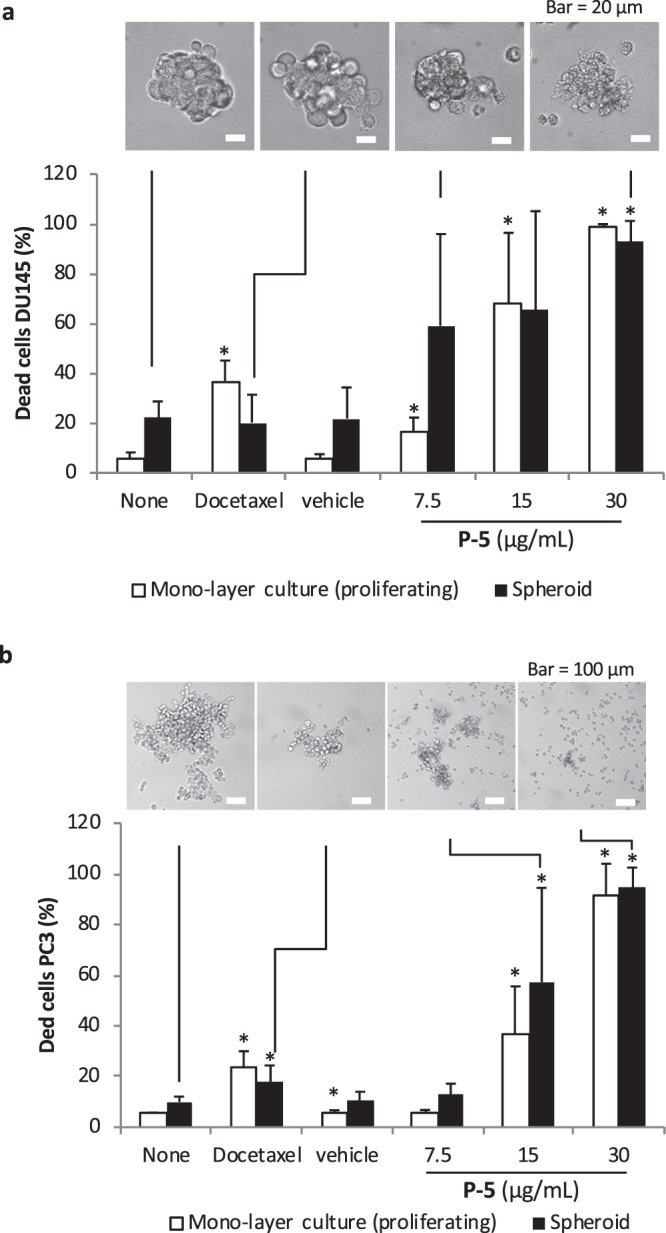
Anticancer activity of **P-5** against PCa cells in mono-layer and spheroid cultures. The cell death (**a**) DU145 and (**b**) PC-3 was determined by staining the cells with Annexin V and DAPI, and the percentage of dead cells was quantified by flow cytometry after dissociating the cell aggregate. Vehicle 0.01% acetic acid (the final concertation of 0.001% in the assay culture). The concertation of docetaxel is 20 ng/mL. The data are expressed as mean ± standard deviation (PC-3; n = 9, DU145; n = 4). Two-tailed, unpaired Student’s t-test was used for data analysis. The statistical significance was determined against the control (None) values. *p < 0.05.

Spherical aggregates or spheroids were prepared by culturing DU145 and PC-3 cells in ultra-low attachment cell culture plates for 24 hours (Fig. 6). The cells showed low expression of Ki-67 protein, indicating slow proliferation or cell dormancy (Fig. S16)^27^. Docetaxel treatment showed little or no effect on the percentage of DU145 cell death, while docetaxel was more active against proliferating cells cultured on a standard cell culture plate (Fig. 6). The drug resistance is likely due to cell dormancy as well as limited diffusion of drug molecules into the spheroids. However, the treatment by **P-5** killed >90% cells in DU145 and PC-3 spheroids. The cell images indicated that the surfaces of cells treated with **P-5** were lysed, and the DU145 spheroids were no longer densely packed, while the cell aggregates treated with docetaxel were rather intact (Fig. 6a). Surprisingly, **P-5** dispersed the PC-3 spheroids and reduced their sizes, indicating that the membrane disruption may also compromise the cell-cell adhesion (Fig. 6b).

## Conclusion

In this study, we demonstrated the molecular design of membrane-active copolymers as a synthetic mimic of ACPs. The copolymers showed anticancer activity against proliferating PCa cells. The copolymers exert their anticancer effects by disrupting the cell membranes. The anticancer activity and selectivity of copolymers could be tuned by altering their cationic and hydrophobic monomer compositions. The anticancer activity of copolymers does not directly reflect the amount of PS lipids exposed on the cell surfaces, and other lipids may be also involved in their mechanism. Importantly, the copolymer killed isolated dormant PCa cells inherently resistant to docetaxel. The copolymer was also effective in killing dormant cells in the *in vitro* tumor spheroid model, resulting in shrinkage or dispersion of *in vitro* tumor spheroid models.

The synthetic polymer platform provides simple, but diverse chemical structures for tuning their physiological properties, providing an approach to improve their efficacy and cancer selectivity. While further specific testing is required, we believe that these traits of polymeric materials will allow rational design and screening of many functional polymers such as degradable polymers^34^, polymer architectures and morphologies such as polymer micelles^35^, and chemical modifications including cancer-homing ligand groups or pro-drug moieities^36–39^, which would lead to identification of clinically potent, selective compounds. In addition, the bio-distribution or systemic toxicity of these polymers are unknown. Synthetic polymers may also induce an immune response, which should be also thoroughly evaluated for clinical translation. Further investigations on therapeutic efficacy and the development of delivery system would be necessary for future clinical implementation. We also envision that anticancer polymers could be used in conjunction with conventional chemotherapies or when conventional chemotherapies fail to treat dormant cancer cells, rather than simply replacing the traditional drugs. Taken together, we believe that the design principle for anticancer polymers demonstrated in this study opens up a new research field to discover new anticancer materials and therapeutics to treat dormant cancer cells.

## Methods

### Polymer synthesis (P-5)

The copolymers were synthesized by RAFT polymerization according to the previous report^40^. For **P-5**, 4-((*tert*-butoxycarbonyl)amino)butyl methacrylate (Boc-ABMA) (7.0 mmol, 1.5 mL of 2 M solution in acetonitrile), ethyl methacrylate (EMA) (3.0 mmol, 1.5 mL of 2 M solution in acetonitrile), 2-cyanoprop-2-yl-dithiobenzoate (1.0 mmol, 221 mg, 10 mole% relative to total amount of monomers), and AIBN (0.1 mmol, 16 mg, 1 mole% relative to total amount of monomers) were mixed in a flask. The oxygen of reaction mixture was removed by nitrogen gas bubbling for 5 minutes, and the reaction solution was stirred at 70 °C for 16 hours. The reaction was stopped by cooling the vial in a dry ice-acetone bath. The solvent was removed by evaporating under reduced pressure. The residue was dissolved in in dichloromethane, and the polymer was isolated by precipitation in excess hexanes twice. The mole percentage of EMA (*MP*_*ethyl*_) was determined by comparing integrated peaks of butylene groups of Boc-ABMA and ethylene groups of EMA in the ^1^H NMR spectrum. The DP was calculated by comparing integrated peaks of phenyl group of chain transfer agent at the polymer ω-end and side chains. The number average molecular weight (*M*_n_) was calculated using the DP, *MP*_*ethyl*_, and the molecular weights of monomers and RAFT agent. Gel permeation chromatography (a calibration curve based on 10 standard samples of poly(methyl methacrylate)): *M*_*n*_ = 2,800 g/mol., *M*_*w*_ = 3,100 g/mol., dispersity (*Đ*) = 1.12. ^1^H NMR (CDCl_3_, 400 MHz) δ: 7.90–7.26 (m, 4H, phenyl end group), 5.25–4.70 (brs, 1H, -N*H*Boc), 4.20–3.83 (m, 2H, side chain of ABMA and EMA -OC*H*_2_-), 3.25–2.92 (brs, 2H, -NC*H*_2_-), 2.15–1.77 (m, 2H, polymer backbone -C*H*_2_-), 1.70–1.45 (m, 4H, side chain of ABMA -NCH_2_C*H*_2_C*H*_2_CH_2_O-), 1.42 (brs, 9H, Boc group), 1.32–1.17 (m, 3H, methyl group of EMA side chain -C*H*_3_), 1.16–0.80 (m, 3H, methyl group of polymer backbone -C*H*_3_). DP (^1^H NMR) = 15.0, *MP*_*ethyl*_ (^1^H NMR) = 30 mole %, *M*_*n*_ (^1^H NMR) = 3,400 g/mol.

Boc-protected **P-5** copolymer (1.00 g) was mixed with methyl 3-mercaptopropionate (MMP) (500 µL), followed by the addition of trifluoracetic acid (TFA) (5 mL). After stirring for 30 min, the TFA was removed by blowing with nitrogen gas. The residue was dissolved in methanol, and the deprotected **P-5** was isolated by precipitating in excess diethyl ether. Subsequently, the precipitate was dissolved in distilled water and lyophilized to yield a light pink fluffy product (0. 94 g, Yield = 90%). ^1^H NMR (methanol-d_4_, 400 MHz) δ: 7.90–7.40 (m, 4H, phenyl end group), 4.20–3.90 (m, 2H, side chain of ABMA and EMA -OC*H*_2_-), 3.10–2.90 (m, 2H, -NC*H*_2_-), 2.20–1.80 (m, 2H, polymer backbone -C*H*_2_-), 1.80–1.65 (m, 4H, side chain of ABMA -NCH_2_C*H*_2_C*H*_2_CH_2_O-), 1.50–1.10 (m, 3H, methyl group of EMA side chain -C*H*_3_), 1.20–0.80 (m, 3H, methyl group of polymer backbone -C*H*_3_). DP (^1^H NMR) = 15.9, *MP*_*ethyl*_ (^1^H NMR) = 31 mole %, *M*_*n*_ (^1^H NMR) = 2,500 g/mol.

### Cell culture

The human prostate cancer cell lines, PC-3 (Cat #: CRL-1435), DU145 (Cat #: HTB-81) and HPrPC (Cat #: PCS-440-010) were obtained from the American Type Culture Collection. All prostate cancer cell lines were routinely grown in RPMI 1640 (Life Technologies, Carlsbad, CA, USA) supplemented with 10% (v/v) fetal bovine serum (Invitrogen), 1% (v/v) penicillin-streptomycin (Invitrogen) and maintained at 37 °C, 5% CO_2_, and 100% humidity. Human dermal microvascular endothelial cells (HDMECs) were obtained from the PromoCell (Heidelberg, Germany) and maintained in endothelial cell growth medium MV2 **(**PromoCell, Heidelberg, Germany) at 37 °C, 5% CO_2_, and 100% humidity. NHDF was obtained from PromoCell.

### Anticancer assays using dormant PC-3 cells

PC-3 cells expressing cell-cycle indicators were incubated with **P-5**, docetaxel, and vehicle (0.01% acetic acid, final concertation of 0.001%) in a culture medium RPMI 1640 containing 0.5% FBS for 48 hours at 37 °C, 5% CO_2_, and 100% humidity. Apoptotic cells or necrotic cells were detected by flow cytometry using APC Annexin V Apoptosis Detection Kit (cat. 640932, BioLegend, San Diego, CA).

### Tumor spheroid model

PC-3 or DU145 cells (5 × 10^4^ cells) were seeded in ultra-low attachment 6-well cell culture plates (Corning #3471) or normal 6-well cell culture plates (Thermo Scientific #140675) in RPMI 1640 containing 10% (v/v) fetal bovine serum, and 48 hours later, Docetaxel or **P-5** was added to the wells (Docetaxel; 20 ng/ml, **P-5**; 7.5, 15, 30 μg/ml). Cell apoptosis/necrosis assay was performed 72 hours after the drug administration.

## Electronic supplementary material

Supplementary Information

## Data Availability

The datasets generated during and/or analyzed during the current study are available from the corresponding author on reasonable request.
