# Author Correction: Anticancer polymers designed for killing dormant prostate cancer cells

**DOI:** 10.1038/s41598-019-53859-y

**Published:** 2019-11-25

**Authors:** Haruko Takahashi, Kenji Yumoto, Kazuma Yasuhara, Enrico T. Nadres, Yutaka Kikuchi, Laura Buttitta, Russell S. Taichman, Kenichi Kuroda

**Affiliations:** 1 Department of Biologic and Materials Sciences, School of Dentistry, University of Michigan, Ann Arbor, MI 48109 USA; 2 Department of Biological Science, Graduate School of Science, Hiroshima University, 1-3-1 Kagamiyama, Higashi-hiroshima, Hiroshima, 739–8526 Japan; 3 Department of Periodontics & Oral Medicine, School of Dentistry, University of Michigan, Ann Arbor, MI 48109 USA; 4 Division of Materials Science, Graduate School of Science and Technology, Nara Institute of Science and Technology, Ikoma, Nara, 630–0192 Japan; 5 Molecular, Cellular and Developmental Biology, University of Michigan, Ann Arbor, MI 48109 USA

Correction to: *Scientific Reports* 10.1038/s41598-018-36608-5, published online 31 January 2019

Laura Buttitta was omitted from the author list in the original version of this Article.

The Acknowledgements section now reads:

“This research was supported by the National Science Foundation (NSF CAREER Award DMR-0845592 to K.K.), the National Cancer Institute (CA093900, CA163124 to R.T.), the Department of Defense (PCRP Idea award W81XW-15-1-0413, to L.B., and W81XWH-14-1-0403 to R.T.), the Prostate Cancer Foundation (Challenge Award 16CHAL05 to R.T.), Grants-in-Aid for Challenging Research (Exploratory, No. 17K19353 to K. Yasuhara) from the Japan Society for the Promotion of Science (JSPS), and Department of Biologic and Materials Sciences, School of Dentistry, University of Michigan. R.T. receives support as the Major McKinley Ash Collegiate Professor. This work was also supported by JSPS Postdoctoral Fellowships for Research Abroad (No. 26-774 to H.T.). We also thank Dr. Robertson Davenport at University of Michigan Hospital for providing RBCs. We thank Dr. Yuji Mishina and Dr. Maiko Omi for help on cell imaging and Dr. Dan Sun for assistance with generating cell lines.”

The Author Contributions section now reads:

“H.T. synthesized and characterized the copolymers, evaluated the cytotoxicity of copolymers to proliferating cancer cells and normal cells, performed LDH leakage assay and cell imaging. K. Yumoto characterized the primary normal cells and dormant cancer cells, evaluated the cytotoxicity of copolymer to normal cells and dormant cancer cells, and prepared cell samples for SEM. K. Yasuhara performed the lipid vesicle assays. E.T.N. synthesized and characterized the copolymers. Y.K. supervised the assays using primary normal cells. L.B. devised and supervised the development of cell lines to monitor dormancy. R.S.T. devised the project and supervised the assays using dormant cancer cells. K.K. devised the project, helped with data analysis, and prepared the manuscript. All authors assisted with editing the manuscript.”

These errors have now been corrected in the PDF and HTML versions of the Article, and in the accompanying Supplementary Information file.

