# Supplementary material for: Anticancer polymers designed for killing dormant prostate cancer cells
Source: Sci Rep. 2019 Jan 31;9:1096. doi: 10.1038/s41598-018-36608-5 (PMC6355926; doi:10.1038/s41598-018-36608-5)
Supplement: Supplementary file 1 — Supplementary Information [file 41598_2018_36608_MOESM1_ESM.docx]

**Supplementary Information**

**Anticancer polymers designed for killing dormant prostate cancer cells**

Haruko Takahashi^1,2†^, Kenji Yumoto^3†^, Kazuma Yasuhara^4^, Enrico T. Nadres^1^, Yutaka Kikuchi^2^, Laura Buttitta^5^ Russell S. Taichman^3^* & Kenichi Kuroda^1^*

1. Department of Biologic and Materials Sciences, School of Dentistry, University of Michigan, Ann Arbor, MI, 48109, USA.
2. Department of Biological Science, Graduate School of Science, Hiroshima University, 1-3-1 Kagamiyama, Higashi-hiroshima, Hiroshima, 739–8526, Japan.
3. Department of Periodontics & Oral Medicine, School of Dentistry, University of Michigan, Ann Arbor, MI, 48109, USA.
4. Division of Materials Science, Graduate School of Science and Technology, Nara Institute of Science and Technology, Ikoma, Nara, 630–0192, Japan.
5. Molecular, Cellular and Developmental Biology, University of Michigan, Ann Arbor, MI 48109, USA.

Haruko Takahashi and Kenji Yumoto contributed equally.

Correspondence and requests for materials should be addressed to R.S.T. (email: rtaich@umich.edu) or K.K. (email: kkuroda@umich.edu)

**Contents**

1. **Experimental**
   1. Materials
   2. Polymer synthesis and characterization.
   3. Cell viability (cytotoxicity) assay using prostate cancer (PCa) cells and HDMECs.
   4. Hemolysis assay.
   5. Annexin V staining and imaging.
   6. LDH leakage assay.
   7. GUVs preparation, data acquisition, and image analysis.
   8. Flow cytometry
   9. PC-3 integrated with cell cycle reporters.
   10. Ki-67 staining.
   11. Annexin V binding to isolated proliferating and dormant PC-3 cells.
   12. Scanning electron microscopy.
   13. Statistical Methods.
2. **Supplementary data**
   1. Characterization of boc-protected and de-protected polymers
   2. Cell viability curves for all cells (C4-2B, DU145, PC-3 and HDMEC) with all polymers after 24 hour incubation with the polymers
   3. Time course of cell viability (DU145) with **P-5**
   4. Hemolysis curves
   5. LDH leakage assay results for other polymers and docetaxel (DU145)
   6. Percentage of released LDH vs. cell viability of cells treated by the copolymers
   7. DU145 cell viability assay for docetaxel
   8. Time dependence of LDH leakage (HDMEC) with P-5
   9. Time dependence of cell viability (HDMEC) with P-5
   10. Analysis of level of Annexin V binding by flow cytometry
   11. Lysis of GUVs
   12. Viability of cells cultured in 0.5% FBS
   13. Ki-67 of isolated dormant PC-3 cells over 48 hours
   14. Cell viability of isolated dormant PC-3 cells over 48 hours
   15. Flow cytometry charts for 1 hour incubation
   16. Expression of Ki-67 protein of cells in spheroids
3. **Experimental**

**1.1. Materials.** Ethanolamine, 4-amino-1-butanol, di-*tert*-butyl dicarbonate, and triethylamine were purchased from Acros Organics. 2,2’-azobisisobutyronitrile (AIBN) and the bee venom toxin melittin (purity > 85 %) were purchased from Sigma-Aldrich Co. LLC. 2-cyanoprop-2-yl-dithiobenzoate was purchased from Strem Chemicals, Inc. Trifluoroacetic acid (TFA) and solvents were purchased from Thermo Fisher Scientific, Inc. The chemicals were used without further purification, with the exception of methacryloyl chloride and ethyl methacrylate (EMA), which were purchased from Acros Organics and distilled before use. ^1^H NMR was performed using a Varian MR400 (400 MHz) and analyzed using VNMRJ 3.2 and MestReNova. Gel permeation chromatography (GPC) analysis was performed using a Waters 1515 HPLC instrument equipped with Waters Styragel (7.8 × 300 mm) HR 0.5, HR 1, and HR 4 columns in sequence and detected by a differential refractometer (RI). Human red blood cells (RBCs) (leukocytes reduced adenine saline added) were obtained from the American Red Cross Blood Services Southeastern Michigan Region and used prior to the out date indicated on each unit. The donors were anonymous and unidentified. The use of cells for hemolysis assay received an approval from the University of Michigan (HUM000054962).

**1.2. Polymer synthesis and characterization.** The synthesis of **P-5** is described in Methods. Other polymers were synthesized by the same polymerization method. The GPC results, degree of polymerization (DP), and mole percentage of ethyl methacrylate (MPethyl) were summarized in Table S1.

**Figure S1. Synthesis of cationic amphiphilic random copolymers. a,** AEMA-EMA copolymers (**P-1**, **P-2**, and **P-3**), and **b,** ABMA-EMA copolymers (**P-4**, **P-5**, and **P-6**).

**P-1 (homopolymer)**. Boc-protected **P-1** was prepared by using 2-((*tert*-butoxycarbonyl)amino)ethyl methacrylate (Boc-AEMA) (4.0 mmol, 4 mL of 1.0 M solution in acetonitrile), 2-cyanoprop-2-yl-dithiobenzoate (0.6 mmol, 132.6 mg, 15 mole% relative to total amount of monomers), and AIBN (0.06 mmol, 60µL of 1.0M solution in acetonitrile, 1 mole% relative to total amount of monomers) (Fig. 1). **De-protected** **P-1**: ^1^H NMR (methanol-d_4_, 400 MHz) δ: 8.00–7.40 (m, 4H, phenyl end group), 4.40–4.00 (brs, 2H, -OC*H*_2_-), 2.40–1.80 (m, 2H, polymer backbone -C*H*_2_-), 1.40–0.80 (m, 3H, methyl group of polymer backbone -C*H*_3_). DP (^1^H NMR) = 15.7, *MP_ethyl_* (^1^H NMR) = 0 mole %, *M_n_* (^1^H NMR) = 2,200 g/mol.

**P-2**. Boc-protected **P-2** was prepared by using 2-((*tert*-butoxycarbonyl)amino)ethyl methacrylate (Boc-AEMA) (3.5 mmol, 802 mg), ethyl methacrylate (EMA) (1.5 mmol, 0.171 mg), 2-cyanoprop-2-yl-dithiobenzoate (1.0 mmol, 221 mg, 20 mole% relative to total amount of monomers), and AIBN (0.05 mmol, 8.2 mg, 1 mole% relative to total amount of monomers) (Fig. 1). **Boc-protected P-2:** *M_n_* (GPC) = 1,800 g/mol., *M_w_* (GPC) = 2,000 g/mol., dispersity (*Đ*) = 1.13. ^1^H NMR (CDCl_3_, 400 MHz) δ: 7.90–7.26 (m, 4H, phenyl end group), 5.4–4.80 (brs, 1H, -N*H*Boc), 4.20–3.80 (m, 2H, side chain of AEMA and EMA -OC*H*_2_-), 3.40–3.20 (brs, 2H, -NC*H*_2_- ), 2.20–1.60 (m, 2H, polymer backbone -C*H*_2_-), 1.42 (brs, 9H, Boc group), 1.32–1.17 (m, 3H, methyl group of EMA side chain -C*H*_3_), 1.16–0.80 (m, 3H, methyl group of polymer backbone -C*H*_3_). DP (^1^H NMR) = 12.3, *MP_ethyl_* (^1^H NMR) = 28 mole %, *M_n_* (^1^H NMR) = 2,600 g/mol. **De-protected** **P-2**: ^1^H NMR (methanol-d_4_, 400 MHz) δ: 8.00–7.40 (m, 4H, phenyl end group), 4.25–4.10 (m, 2H, side chain of AEMA and EMA -OC*H*_2_-), 2.16–1.82 (m, 2H, polymer backbone -C*H*_2_-), 1.50–1.20 (m, 3H, methyl group of EMA side chain -C*H*_3_), 1.20–0.80 (m, 3H, methyl group of polymer backbone -C*H*_3_). DP (^1^H NMR) = 12.8, *MP_ethyl_* (^1^H NMR) = 28 mole %, *M_n_* (^1^H NMR) = 1,800 g/mol.

**P-3**. Boc-protected **P-3** was prepared by using 2-((*tert*-butoxycarbonyl)amino)ethyl methacrylate (Boc-AEMA) (3.0 mmol, 0.688 mg), ethyl methacrylate (EMA) (3.0 mmol, 342 mg), 2-cyanoprop-2-yl-dithiobenzoate (1.2 mmol, 266 mg, 20 mole% relative to total amount of monomers), and AIBN (0.06 mmol, 9.9 mg, 1 mole% relative to total amount of monomers) (Fig. 1). **Boc-protected P-3:** *M_n_* = 1,700 g/mol., *M_w_* = 1,900 g/mol., dispersity (*Đ*) = 1.10. ^1^H NMR (CDCl_3_, 400 MHz) δ: 7.90–7.26 (m, 4H, phenyl end group), 5.25–4.70 (brs, 1H, -N*H*Boc), 4.20–3.83 (m, 2H, side chain of AEMA and EMA -OC*H*_2_-), 3.25–3.00 (brs, 2H, -NC*H*_2_- ), 2.20–1.60 (m, 2H, polymer backbone -C*H*_2_-), 1.42 (brs, 9H, Boc group), 1.30–1.20 (m, 3H, methyl group of EMA side chain -C*H*_3_), 1.20–0.80 (m, 3H, methyl group of polymer backbone -C*H*_3_). DP (^1^H NMR) = 11.9, *MP_ethyl_* (^1^H NMR) = 45 mole %, *M_n_* (^1^H NMR) = 2,300 g/mol. **De-protected** **P-3**. ^1^H NMR (methanol-d_4_, 400 MHz) δ: 7.90–7.40 (m, 4H, phenyl end group), 4.25-3.83 (m, 2H, side chain of ABMA and EMA -OC*H*_2_-), 2.40–1.80 (m, 2H, polymer backbone -C*H*_2_-), 1.50 (brs, 3H, methyl group of EMA side chain -C*H*_3_), 1.20–0.80 (m, 3H, methyl group of polymer backbone -C*H*_3_). DP (^1^H NMR) = 13.0, *MP_ethyl_* (^1^H NMR) = 40 mole %, *M_n_* (^1^H NMR) = 1,800 g/mol.

**P-4 (homopolymer)**. Boc-protected **P-4** was prepared by using 4-((*tert*-butoxycarbonyl) amino)butyl methacrylate (Boc-ABMA) (1.0 mmol, 0.5 mL of 2 M solution in acetonitrile), 2-cyanoprop-2-yl-dithiobenzoate (0.1 mmol, 22 mg, 10 mole% relative to total amount of monomers), and AIBN (0.01 mmol, 2 mg, 1 mole% relative to total amount of monomers) (Fig. 1). **Boc-protected P-4:** *M_n_* = 2,200 g/mol., *M_w_* = 2,900 g/mol., dispersity (*Đ*) = 1.32. ^1^H NMR (CDCl_3_, 400 MHz) δ: 7.90–7.26 (m, 4H, phenyl end group), 5.60–5.00 (brs, 1H, -N*H*Boc), 4.20–3.83 (m, 2H, side chain of ABMA -OC*H*_2_-), 3.40–3.20 (brs, 2H, -NC*H*_2_- ), 2.15–1.77 (m, 2H, polymer backbone -C*H*_2_-), 1.60 (m, 4H, side chain of ABMA -NCH_2_C*H*_2_C*H*_2_CH_2_O-), 1.42 (brs, 9H, Boc group), 1.32–1.20 (m, 3H, methyl group of EMA side chain -C*H*_3_), 1.15–0.80 (m, 3H, methyl group of polymer backbone -C*H*_3_). DP (^1^H NMR) = 14.1, *MP_ethyl_* (^1^H NMR) = 0 mole %, *M_n_* (^1^H NMR) = 3,800 g/mol. **De-protected** **P-4**: ^1^H NMR (methanol-d_4_, 400 MHz) δ: 7.90–7.40 (m, 4H, phenyl end group), 4.25–3.90 (m, 2H, side chain of ABMA and EMA -OC*H*_2_-), 3.10–2.80 (m, 2H, -NC*H*_2_-), 2.20–1.80 (m, 2H, polymer backbone -C*H*_2_-), 1.80 (m, 4H, side chain of ABMA -NCH_2_C*H*_2_C*H*_2_CH_2_O-), 1.50–0.80 (m, 3H, methyl group of polymer backbone -C*H*_3_). DP (^1^H NMR) = 13.9, *MP_ethyl_* (^1^H NMR) = 0 mole %, *M_n_* (^1^H NMR) = 2,400 g/mol.

**P-6**. Boc-protected **P-6** was prepared by using 4-((*tert*-butoxycarbonyl) amino)butyl methacrylate (Boc-ABMA) (5.0 mmol, 1.29g), ethyl methacrylate (EMA) (5 mmol, 0.63ml), 2-cyanoprop-2-yl-dithiobenzoate (1.5 mmol, 332 mg, 15 mole% relative to total amount of monomers), and AIBN (0.1 mmol, 16 mg, 1 mole% relative to total amount of monomers) (Fig. 1). **Boc-protected P-6:** *M_n_* = 2,100 g/mol., *M_w_* = 2,400 g/mol., dispersity (*Đ*) = 1.12. ^1^H NMR (CDCl_3_, 400 MHz) δ: 7.90–7.30 (m, 5H, phenyl end group), 5.20–4.60 (brs, 1H, -N*H*Boc), 4.20–3.80 (m, 2H, side chain of ABMA and EMA -OC*H*_2_-), 3.20–3.00 (brs, 2H, -NC*H*_2_- ), 2.20–1.70 (m, 2H, polymer backbone -C*H*_2_-), 1.70–1.45 (m, 4H, side chain of ABMA -NCH_2_C*H*_2_C*H*_2_CH_2_O-), 1.40 (brs, 9H, Boc group), 1.20–1.10 (m, 3H, methyl group of EMA side chain -C*H*_3_), 1.10–0.80 (m, 3H, methyl group of polymer backbone -C*H*_3_). DP (^1^H NMR) = 14.2, *MP_ethyl_* (^1^H NMR) = 50.8 mole %, *M_n_* (^1^H NMR) = 2,800 g/mol. **De-protected** **P-6**: ^1^H NMR (methanol-d_4_, 400 MHz) δ: 7.90–7.40 (m, 4H, phenyl end group), 4.20-3.80 (m, 2H, side chain of ABMA and EMA -OC*H*_2_-), 3.10–2.80 (m, 2H, -NC*H*_2_-), 2.20-1.80 (m, 2H, polymer backbone -C*H*_2_-), 1.80–1.65 (m, 4H, side chain of ABMA -NCH_2_C*H*_2_C*H*_2_CH_2_O-), 1.50–1.10 (m, 3H, methyl group of EMA side chain -C*H*_3_), 1.10–0.80 (m, 3H, methyl group of polymer backbone -C*H*_3_). DP (^1^H NMR) = 16.6, *MP_ethyl_* (^1^H NMR) = 48 mole %, *M_n_* (^1^H NMR) = 2,500 g/mol.

**1.3. Cell viability (cytotoxicity) assay using prostate cancer (PCa) cells and HDMECs.**

PCa and HDMEC cells were seeded at 1 × 10^4^ cells/well in 96-well sterile flat-bottom polystylene cell culture plate and cultured for 24 h. Then cells were exposed to a serial concentration of polymers (**P-1** to **P-6**) for 24, 48 or 72 hours. The bee venom toxin melittin was also tested as reference standard. 0.01 % acetic acid was used as vehicle. After 24 hours treatment by polymers or vehicle, the cell morphologies were observed by a microscope (Carl Zeiss Axio Observer 7). Then, medium containing polymers were removed and cells were washed with PBS. The cells’ viability after exposure to the polymers was measured using Cell Counting Kit-8 (CCK-8) (Dojindo) or Cell Proliferation Kit II (XTT, Roche Applied Sciences) by measuring the amount of the metabolized formazan at 450 nm using Varioskan Flash microplate reader (Thermo Fisher) following standard protocols from products. The percentage of cells viability was determined relative to 0.01% acetic acid (100% cell viability). Each assay was independently repeated three times using different stock solutions in triplicate on different days.

**1.4. Hemolysis assay.** Human red blood cells from healthy donors (RBCs; 1 mL) were suspended in 9 mL of PBS buffer (pH 7.4) and centrifuged at 660 × g for 5 min. The supernatant was removed by pipetting and RBCs were re-suspended in PBS. This procedure was repeated two additional times. The number of RBCs in resulting suspension was counted by a counting chamber and diluted in PBS to give 3.0 × 10^8^ cells/mL as a final concentration. After serial dilutions of polymers (**P-1** to **P-6**) were prepared on a 96-well sterile round-bottom polypropylene plate, the RBC suspension (90 μL) was added and incubated at 37°C with orbital shaking (180 rpm). Triton X-100 (0.1% v/v in water) was used as the positive lysis control and 0.01% acetic acid were used as negative control. The bee venom toxin melittin was also tested as reference standard. After incubation for 1 h, the plate was centrifuged at 1000 × g for 5 min and supernatant (6 μL) from each well was diluted with PBS buffer (100 μL) in a 96-well sterile flat-bottom polystylene plate. The absorbance of the released hemoglobin at 415 nm was measured using Varioskan Flash microplate reader (Thermo Fisher). The percentage of hemolysis was calculated relative to the positive control Triton X-100 (100%) and negative control 0.01% acetic acid (0%). The HC_50_ was defined as the polymer concentration causing 50% hemolysis. The HC_50_ or hemolysis% at highest concentration if the hemolysis% showed below 50% was reported. Each hemolysis assay was independently repeated two or three times using different stock solutions in triplicate on different days.

**1.5. Annexin V staining and imaging.** PCa and HDMEC cells were seeded at 1 × 10^4^ cells/dish on sterile glass-bottom dish (glass diameter= 10 mm) and cultured for 24 h. Then 5 µL of PE Annexin V solution (BD Bioscience) was added to each glass bottom dishes and incubated for 15 min at 37°C as following standard protocols from a product. The cells were washed with PBS and observed by a microscope with lenses of 20× magnification (Olympus Eclipse TE300).

**1.6. LDH leakage assay.** The DU145 cells were seeded at 1 × 10^4^ cells/well in 96-well sterile flat-bottom polystylene cell culture plate and cultured for 24 h. Then cells were exposed to a serial concentration of polymers (**P-1** to **P-6**) for 24 hours. The bee venom toxin melittin was also tested as reference standard. 0.01 % acetic acid was used as vehicle. After 24 hours treatment, 10 µL of the Lysis Buffer of Cytotoxicity LDH assay Kit-WST (Dojindo) was added to only 0.01% acetic acid wells (100% cell lysis) and incubated for 30 min at 37°C as following standard protocols from a product. Then, 100 µL of the Working Solution to all wells and incubated for 30 min at room temperature. To stop the reaction, 50 µL of the Stop Solution was added and absorbance at 490 nm was measured by Varioskan Flash microplate reader (Thermo Fisher). The percentage of LDH leakage was determined relative to 0.01% acetic acid (100% cell lysis). Each assay was independently repeated three times using different stock solutions in triplicate on different days.

**1.7. GUV preparation, data acquisition, and image analysis.** Giant vesicles were prepared by the gentle hydration method as follows. Thin lipid films composed of DOPC alone and DOPC/DSPC (65:35) were hydrated with 200 mM sucrose in 1 mM HEPES (pH=7.4) buffer at room temperature for overnight. 10 µL of obtained vesicular dispersions were diluted with 90 µL of 1 mM HEPES (pH=7.4) buffer containing 200 mM glucose to monitor the leakage of entrapped sucrose using phase contrast microscope. Due to the difference of the reflective indexes of inner and outer phases, the inside of the vesicle displays black contrast. Microscopic observation was performed using an inverted microscope (Olympus IX71, Tokyo, Japan) equipped with a 20x objective lens in phase contrast mode. Microscopic images were recorded using a CMOS camera (ORCA-Flash 2.8, Hamamatsu Photonics, Hamamatsu, Japan). To record the time-lapse response of the GUVs against vesicles, the GUV dispersion was placed on a channel slide (ibidi µ-Slide VI, Martinsried, Germany). The movie of the GUVs response was recorded approximately 2 mm away from the injection port for the polymer solution (Movies S1 and S2). Obtained images were analyzed using an ImageJ software to estimate the number of the GUVs and corresponding diameter.

**1.8. Flow cytometry.** The flow cytometric analyses and fluorescence-activated cell sorting (FACS) were performed on a FACS Aria dual-laser flow cytometer (Becton Dickinson, Franklin Lakes, NJ) and data were analyzed with DIVA software (Becton Dickinson). BD cytometer setup & tracking beads (BD Biosciences, Cat #: 642412) were used for the daily instrument standardization and validation. Sorting calibration was performed before each sort by drop-delay using Accudrop beads (BD Biosciences, Cat #: 345249). Cells were detached using 0.25% Trypsin-EDTA expect for HPrEC. HPrEC was detached using Trypsin-EDTA for Primary Cells (ATCC® PCS-999-003) and the Trypsin Neutralizing Solution (ATCC PCS-999-004) according to the ATCC recommendation.

**1.9. PC3 integrated with cell cycle reporters.** To develop a method to identify dormant or slow-cycling prostate cancer cells, we transduced a human prostate cancer cell line, PC-3 with lentiviruses containing the fluorescent ubiquitination-based cell cycle reporters (Oki et al, Sci Rep. 2014). Both of the CDT1-mCherry reporter (pMXs-mCherry-hCdt1(30/120) and the p27 cyclin-dependent kinase inhibitor protein –Venus reporter (pMXs-IP-mVenus-p27) were packaged into lentivirus at the University of Michigan Vector Core Facility. PC-3 cells infected with both of the lentiviral reporters were selected for 7 days in RPMI medium containing 10 μg/ml puromycin. To isolate the cells which are successfully integrated with both reporters, Venus and mCherry double positive cells were sorted by flow cytometry. Isolated PC-3 Venus mCherry cells were cultured in the RPMI containing 10% FBS. P27-Venus is upregulated upon entry into quiescence and is tagged for degradation by the Kip1 ubiquitination-promoting complex (KPC) in late G1 and the Skp2 ubiquitin ligase in the G1-S transition. Therefore, this reporter is high during G0, but low upon G1 entry and the G1-S transition (Oki et al, Sci Rep. 2014). The Cdt1-mCherry reporter is high during G0 and G1, but degraded during S phase by Skp2-dependent degradation (Oki et al, Sci Rep. 2014). Together, these two reporters can be used to identify dormant cells.

**1.10. Ki-67 staining.** Cells were fixed with cold 70% ethanol, and stained with an APC conjugated anti-human Ki-67 antibody (Cat #: 350513, Biolegend) in PBS containing 2% FBS for 30 minutes at room temperature. Ki-67 expression levels were examined using a FACS Aria dual-laser flow cytometer.

**1.11. Annexin V binding to isolated proliferating and dormant PC-3 cells.** The cell-cycle reporter integrated cells were sorted by flow cytometry, and the proliferating (p27(-)/Cdt1(-)) and dormant PC-3 (p27(+)/Cdt1(+)) cells were incubated with Annexin V-APC. The level of Annexin V binding was determined by flow cytometry.

**1.12. Scanning electron microscopy.** The effect of **P-5** on the cell morphologies of dormant cancer cells was examined using AMRAY 1910 Field Emission Scanning Electron Microscope (FE-SEM). The cell cycle reporter-integrated PC-3 cells were cultured in a culture medium (RPMI1640) with 0.5% FBS for 48 hours, and dormant cells (p27 and Cdt1 positive) were isolated by sorting using a FACS Aria dual-laser flow cytometer (Becton Dickinson, Franklin Lakes, NJ). The isolated cells (1 × 10^5^ cells) were seeded in a well of culture chambers (Lab-Tek II Chambered Coverglass Culture Area 155379) and cultured in 1 ml of culture medium with 0.5% FBS for 48 hours. The cells were treated by docetaxel at 20 ng/mL or **P-5** at 30 µg/mL. After 1 hour incubation, the cells were washed by PBS and fixed overnight by 2.5% glutaraldehyde in 0.1 M Sorensen’s phosphate buffer, pH7.4, and then rinsed three times for 15 minutes each with 0.1 M Sorensen’s buffer. The samples were further fixed for one hour at 4^o^C in 1 % osmium tetroxide in 0.1 M Sorensen’s buffer and rinsed three times with 0.1 M Sorensen’s phosphate buffer, 15 minutes each. The fixed samples were dehydrated for 10 minutes each in 30, 50, 70, 80, 90, 95 and two changes of 100 % ethanol, and then 2 changes of Hexamethyldisilizane (HMDS) for 10 minutes each. All but a few drops of HMDS were removed, and the samples were air-dried in the hood overnight. The sample coverslip was mounted on aluminum stub with conductive paint, left overnight for outgassing of adhesive, and coated with gold (sputter coating). The SEM images were obtained using an Amray 1910 FE-SEM.

**1.13. Statistical Methods.** All numerical data are expressed as mean ± standard deviation unless specified otherwise. Two-tailed, unpaired Student’s t-test was used for data analysis, with p < 0.05 considered to be statistically significant.

1. **Supplementary Data**
   1. **Characterization of boc-protected and de-protected polymers**

**Table S1. Characterization of boc-protected and de-protected polymers**

| Polymer | Boc-protected polymer | | | | | | De-protected polymer | | |
| --- | --- | --- | --- | --- | --- | --- | --- | --- | --- |
|  | *M_n_* (GPC) (g/mol.) | *M_w_* (GPC) (g/mol.) | *M_n_* (NMR) (g/mol.) | *Đ* | DP (NMR) | *MP_ethyl_* (NMR) (mol.%) | DP (NMR) | *MP_ethyl_* (NMR) (mol.%) | *M_n_*  (NMR)  (w/o TFA) |
| **P-1** | - | - | - | - | - | 0 | 15.7 | 0 | 2200 |
| **P-2** | 1800 | 2000 | 2600 | 1.13 | 12.3 | 28 | 12.8 | 28 | 1800 |
| **P-3** | 1700 | 1900 | 2300 | 1.10 | 11.9 | 45 | 13.0 | 40 | 1800 |
| **P-4** | 2200 | 2900 | 3800 | 1.32 | 14.1 | 0 | 13.9 | 0 | 2400 |
| **P-5** | 2800 | 3100 | 3400 | 1.12 | 15.0 | 30 | 15.9 | 31 | 2500 |
| **P-6** | 2100 | 2400 | 2800 | 1.12 | 14.2 | 51 | 16.6 | 48 | 2500 |

- 1. **Cell viability curves for all cells (C4-2B, DU145, PC-3, HDMEC, NHDF, and HPrE) after 24 hour incubation with the polymers**

Figure S2. Cell viability of C4-2B (a), DU145 (b), PC-3 (c), HDMEC (d), NHDF (e), and HPrEC (f) cells after 24 fours polymer and melittin treatment.

- 1. **Time course of cell viability (DU145) with P-5**

Figure S3. Time course of cell viability on DU145 treated by **P-5** polymer.

- 1. **Hemolysis curves**

Figure S4. Hemolysis curves for all the polymers and melittin.

- 1. **LDH leakage assay results for polymers and docetaxel (DU145)**

Figure S5. LDH leakage of DU145 cells after 24 fours treatment with polymers and melittin (a) and docetaxel (b).

- 1. **Percentage of released LDH vs. cell viability of cells treated by the copolymers**

Figure S6. Correlations between released LDH vs. cell viability with copolymers (a, b) and docetaxel (c).

- 1. **DU145 cell viability assay for docetaxel**

Figure S7. Time course of cell viability on DU145 treated by docetaxel.

- 1. **Time dependence of LDH leakage (HDMEC) with P-5**

Figure S8. LDH leakage of HDMEC after 24 four and 72 hour treatment with **P-5** polymer.

- 1. **Time dependence of cell viability (HDMEC) with P-5**

Figure S9. Time dependence of cell viability on HDMEC treated by **P-5** polymer.

- 1. **Analysis of level of Annexin V binding by flow cytometry**

Figure S10. Level of Annexin V-PE staining. The cells were detached from cell culture plates

and stained by Annexin V-PE expect HPrEC which was stained by Annexin V-APC.

- 1. **Lysis of GUVs**

Figure S11. Images of GUVs treated by **P-5** in varied polymer concentrations.

- 1. **Viability of cells cultured in 0.5% FBS**

Figure S12. Cell viability of PC-3 cells in 0.5% FBS cell culture medium.

- 1. **Ki-67 of isolated dormant PC-3 cells over 48 hours**

Figure S13. Expression of endogenous proliferation Ki-67 protein in isolated PC-3 cells over 48 hours.

- 1. **Cell viability of isolated dormant PC-3 cells over 48 hours**

Figure S14. Cell proliferation over 48 hours in a 0.5% FBS medium. **a**, proliferative cells (p27(-) /Cdt1(-)), and **b**, dormant cells (p27(+) /Cdt1(+)).

- 1. **Flow cytometry charts for 1 hour incubation**

Figure S15. Flow cytometric analysis of treated isolated dormant PC-3 cells. The dormant PC-3 cells were incubated with **P-5** at 30 µg/mL, docetaxel at 20 ng/mL, or vehicle (0.01% acetic acid) for one hour in a 0.5% FBS culture medium.

- 1. **Expression of Ki-67 protein of cells in spheroids**

Figure S16. Expression of endogenous proliferation Ki-67 protein in prostate cancer spheroid cultures (**a**, DU145 and **b**, PC-3) .
